# Social and economic impacts of congenital Zika syndrome in Brazil: Study protocol and rationale for a mixed-methods study

**DOI:** 10.12688/wellcomeopenres.14838.2

**Published:** 2019-09-11

**Authors:** Hannah Kuper, Tereza Maciel Lyra, Maria Elisabeth Lopes Moreira, Maria do Socorro Veloso de Albuquerque, Thália Velho Barreto de Araújo, Silke Fernandes, Mireia Jofre-Bonet, Heidi Larson, Ana Paula Lopes de Melo, Corina Helena Figueira Mendes, Martha Cristina Nunes Moreira, Marcos Antonio Ferreira do Nascimento, Loveday Penn-Kekana, Camila Pimentel, Marcia Pinto, Clarissa Simas, Sandra Valongueiro

**Affiliations:** 1 International Centre for Evidence in Disability, Clinical Research Department, London School of Hygiene & Tropical Medicine, London, UK; 2 Aggeu Magalhães Institute, FIOCRUZ/PE, Recife, Brazil; 3 Faculty of Medicine, University of Pernambuco, Recife, Brazil; 4 Fernando Figueira Maternal and Children's Institute, Rio de Janeiro, Brazil; 5 Department of Social Medicine, Federal University of Pernambuco, Recife, Brazil; 6 Postgraduate Programme in Public Health, Federal University of Pernambuco, Recife, Brazil; 7 Faculty of Public Health and Policy, London School of Hygiene & Tropical Medicine, London, UK; 8 Department of Economics, City University London, London, UK; 9 Department of Infectious Disease Epidemiology, London School of Hygiene & Tropical Medicine, London, UK; 10 Public Health Department, Federal University of Pernambuco, Recife, Brazil

**Keywords:** Zika, Congenital Zika Syndrome, Economic, Social, Depression, Anxiety, Brazil

## Abstract

Global concern broke out in late 2015 as thousands of children in Brazil were born with microcephaly, which was quickly linked to congenital infection with Zika virus (ZIKV). ZIKV is now known to cause a wider spectrum of severe adverse outcomes—congenital Zika syndrome (CZS)—and also milder impairments. This study aimed to explore the social and economic impacts of CZS in Brazil. Data was collected through mixed methods across two settings: Recife City and Jaboatão dos Guararapes in Pernambuco State (the epicentre of the epidemic), and the city of Rio de Janeiro (where reports of ZIKV infection and CZS were less frequent). Data was collected May 2017-January 2018. Ethical standards were adhered to throughout the research. In-depth qualitative interviews were conducted with: mothers and other carers of children with CZS (approximately 30 per setting), pregnant women (10-12 per setting), men and women of child-bearing age (16-20 per setting), and health professionals (10-12 per setting). Thematic analysis was undertaken independently by researchers from at least two research settings, and these were shared for feedback.

A case-control study was undertaken to quantitatively explore social and economic differences between caregivers of a child with CZS (cases) and caregivers with an unaffected child (controls). We aimed to recruit 100 cases and 100 controls per setting, from existing studies. The primary caregiver, usually the mother, was interviewed using a structured questionnaire to collect information on: depression, anxiety, stress, social support, family quality of life, health care and social service use, and costs incurred by families. Multivariable logistic regression analyses were used to compare outcomes for cases and controls. Costs incurred as a result of CZS were estimated from the perspective of the health system, families and society. Modelling was undertaken to estimate the total economic burden of CZS from those three perspectives.

## Introduction

The Zika virus (ZIKV) epidemic hit Brazil in early 2015, coinciding with a time of political and economic crisis for the country ^1^. By the end of 2015, thousands of children had been born with microcephaly, which was quickly linked to ZIKV ^2^. It is now clear that ZIKV is also associated with a wider spectrum of severe adverse outcomes, collectively termed congenital Zika syndrome (CZS), including the following five distinctive features ^3^:

- Severe microcephaly in which the skull has partially collapsed
- Decreased brain tissue with a specific pattern of brain damage, including subcortical calcifications
- Damage to the back of the eye, including macular scarring and focal pigmentary retinal mottling
- Congenital contractures, such as clubfoot or arthrogryposis
- Hypertonia restricting body movement soon after birth

CZS is therefore not limited to children with microcephaly, and the criteria for determining CZS are still being refined and will increase in precision as more phenotypic data becomes available. Confirmation of CZS has also been made more complex by the fact that evidence of ZIKV exposure in the mother is not always available, yet needed for the CZS diagnosis. Nevertheless, by early 2018, 3,149 cases of CZS were confirmed, with a further 447 probable cases and 2,795 under investigation ^4^. The vast majority of children with CZS had been born by early 2016 and only sporadic cases were reported thereafter. Overall, estimates from 8 studies suggest that 2.3% (range 1.0–5.3%) of pregnancies of ZIKV-infected women resulted in the birth of a child with microcephaly ^5^, with a recent study showing an even higher proportion (5.8%) ^6^.

Parallels with maternal infection with cytomegalovirus or rubella may be instructive, as both can lead to microcephaly, but far more commonly to a broad range of milder impairments (e.g. hearing, visual, intellectual) which are not always apparent in newborns ^7^. Similarly, congenital ZIKV infection is now also associated with other abnormalities, aside from CZS, including brain injuries, hearing, visual and musculoskeletal abnormalities of variable severity ^8– 11^, with one study showing a ratio of clinical/brain imaging abnormalities to microcephaly of approximately 11:1 ^11^. ZIKV infection in pregnancy is also linked to fetal death, placental insufficiency, and intrauterine growth restriction ^12^.

The serious consequences of congenital infection with ZIKV meant that the epidemic attracted high levels of national and international attention. A major focus of the response to the ZIKV epidemic at the local/regional level has been on the elimination of the vector, while national and international efforts have also focussed on the development of vaccines, point-of-care diagnosis, and treatment. Avoidance of pregnancy was also encouraged during the peak of the epidemic, and new data suggests that the number of births did fall in Brazil, potentially due to postponement of pregnancy and an increase in abortions ^13^. However, the areas where ZIKV was most prevalent are also those where contraceptive availability is most limited ^14^, opening discussion about provision of family planning, and even abortion, which is currently illegal in Brazil ^15^.

By contrast, the impact of the ZIKV epidemic on mothers, families and society has received little attention. The social impacts of CZS are likely to be large, given its link with severe and broad-ranging disabilities, particularly for the mother who are almost always the main carer. Affected babies may be irritable and more difficult to care for, as one mother said: *“For every ten minutes of sleep, she cries for an hour”* ^16^. We know that in other settings, mothers of severely disabled children are likely to experience stress and depression and potentially marital breakdown ^17, 18^, and this may be more pronounced for CZS given the unknown nature of the condition and its prognosis ^19^. The adverse outcomes among children with CZS may be ameliorated by early intervention, specialist care and provision of support for mothers, although by-and-large these have not been prioritized in the response, with a few notable exceptions ^20^. Development of appropriate services for caregivers and children will require a more detailed understanding of the impact of CZS ^19, 21^. Social impacts will potentially extend beyond the mothers and other caregivers directly affected. For instance, impacts on reproductive decision-making of women in affected areas are likely and need to be investigated, as well as for men, although they are rarely considered ^22^. There may also be important negative psychological impacts on pregnant women ^23^, who may experience high levels of stress due to the possibility of ZIKV infection. Impacts on health professionals are also important to consider, as they are relied upon to provide information and care to affected children and those concerned about ZIKV, yet at the onset of the epidemic little was known about the disease and its consequences.

Economic consequences of CZS are also likely to be important. Parents of the children with CZS will incur direct (e.g. payment for medicines) and indirect costs (e.g. lost productivity). This impact is potentially exacerbated by the fact that ZIKV disproportionately affects poor people ^24^, who are less able to cope with this economic burden. The government will also spend money for the provision of health and social services for affected children and their caregivers. It is important to calculate these economic costs of ZIKV, so that decision makers can understand the economic impact of each case and be able to benchmark this against other diseases, and prepare appropriate budgets. Early estimates suggest that each case of microcephaly incurs direct medical costs of $91,102 and $28,818 per lifetime for Latin America and the Caribbean, respectively ^25^. However, these estimates were based on extrapolations and did not include indirect costs, warranting a more comprehensive investigation. Furthermore, understanding the social and economic impact of CZS will help decision makers to tailor responses by the health and social services.

The ZIKV epidemic is the fourth Public Health Emergency of International Concern declared by the WHO, since 2009. The global community must be better equipped to alleviate the impact of epidemics in the future, whether in terms of ZIKV epidemics in new areas or epidemics of different diseases. Development of a set of approaches to measure social and economic impact at the individual and societal levels that can be used in other settings and for other conditions is therefore needed.

As a consequence of these gaps in evidence, the aim of this research study is to describe the social and economic impacts of CZS in Brazil. Specific objectives are to:

1. Describe the social and economic impact of CZS at the mother, family and societal levels.
2. Understand the beliefs and attitudes concerning contracting ZIKV when pregnant, and the impact on family planning, sexual and reproductive health, and unsafe abortions.
3. Understand the consequences of CZS for health services and systems, and other social provision.
4. Identify key learning lessons of how to mitigate the social and economic impacts of CZS.
5. Develop tools to measure social and economic impact that can be used in future epidemics of ZIKV or other diseases.

## Protocol

### Overview and setting

A mixed-methods study was used to assess the social and economic impacts of ZIKV, implemented by a multi-disciplinary team, including researchers from Brazil and the UK. Qualitative and quantitative (including economic) data were collected. Two contrasting sites were selected where work was ongoing and the teams had good access to mothers of children with CZS. The first was Recife City and Jaboatão dos Guararapes, in the State of Pernambuco in Northeast Brazil. The Northeast region has a high number of suspected and confirmed cases of CZS, and is considered the epi-centre of the epidemic ^4^. For contrast, the second site was Rio de Janeiro City, in the State of Rio de Janeiro, where symptomatic ZIKV was less prevalent and reports of CZS far lower.

### Qualitative data collection

The qualitative component of this research aimed to: understand the social and economic impacts of CZS at the mother and household level; describe the impacts of ZIKV on the beliefs and attitudes of pregnant women and on family planning decision-making; and explore the sources and adequacy of information on ZIKV and CZS given to women, their families, and healthcare workers. To generate this information, in-depth qualitative interviews were conducted with the following groups of respondents in both Recife and Rio de Janeiro:

1. Mothers and other caregivers (e.g. father, grandmother) of children with CZS were recruited from the quantitative studies in Recife and Rio de Janeiro (described below). We aimed to recruit 15 mothers and 15 other caregivers per setting, and these were not necessarily paired (e.g. mother and father of same child). We purposively sampled participants to identify a range of subjects, with respect to: severity of syndrome, age of the child, age of the caregiver, ethnicity of the mother, and socio-economic status. The sample was restricted to people living in the urban areas. In Recife, interviews with the mothers and caregivers were conducted in person in their home. In Rio de Janeiro, interviews with the mothers were conducted at Fernandes Figueira Institute (IFF/Fiocruz) and at other healthcare settings.
2. Women and men of reproductive age were recruited through primary healthcare facilities in the two settings, as they presented for routine health check-ups in Rio de Janeiro and in Recife city and Jaboatão dos Guararapes (a city in the metropolitan area of Recife). We aimed to include 8–10 men and 8–10 women per setting.
3. Pregnant women were recruited through primary healthcare facilities in Recife city and Jaboatão dos Guararapes, as they presented for routine ante-natal appointments. In Rio de Janeiro, pregnant women were recruited through the ongoing “Vertical Exposure to Zika Virus and Its Consequences for Child Neurodevelopment: Cohort Study in Fiocruz/IFF” (ClinicalTrials.gov Identifier: NCT03255369). We did not differentiate whether the women were Zika positive or negative during pregnancy ^1^. We aimed to include 10–12 pregnant women per site.
4. Healthcare professionals were recruited at both the hospital and primary healthcare level, and we aimed to include a range of specialists (e.g. ophthalmologists, physiotherapists) per setting, as well as a clinical epidemiologist at each site. Health agents were not included in the sample. We aimed to include 10–12 healthcare professionals per site.

Interviews were conducted between March and November, 2017. The target number of interviews was indicative, and would be extended if data saturation was not reached. Interviews were conducted by trained interviewers using a topic guide, which had been developed by the research team and pilot tested and adapted where necessary ( Supplementary File 1). A total of three interviewers were used in Recife and four in Rio de Janeiro. All interviewers were female Brazilian social scientists from the local region, who were either already experienced, or had undergone training by senior researchers in the group, which included role-play exercises and practice with the interview guides. All interviews were recorded on a digital recorder. In addition, the interviewers took notes which were shared with the research team. The interviewers came together regularly with the senior researchers to discuss key findings, difficulties, and any changes needed in the interview guides.

### Quantitative data collection

A case-control study was undertaken to collect quantitative data to explore the differences between mothers with a child affected by CZS, in terms of social and economic variables.

We aimed to recruit 100 cases and 100 controls per setting, which would provide the power to detect an OR of 2.6 in each site for the association between depression and CZS, assuming 95% confidence, 80% power and a prevalence of depression of 15% in unaffected mothers ^26^. Across the two samples (i.e. 200 cases and 200 controls), the sample size would be adequate to detect an OR of 2.05 for the same association.

In Recife, the source of most of the cases and all the controls was an existing case-control study, initiated in January 2016. Methods of the case-control study have been published in full ^27^. Briefly, cases were children born with microcephaly (head circumferences < 2 SD than the mean) in one of eight public maternity hospitals in Recife. Controls were children born in the same hospitals, but without microcephaly and without neurological or other health problems (determined from transfontanellar ultrasonography, and through physical examination by the study neonatologist), with both examinations performed soon after birth. Controls were matched to cases on the basis of expected date of delivery and place of mother’s residence (by Health Region). During the follow-up interview parents of control children were asked 3–4 age-appropriate questions to assess whether there were any apparent developmental delays (based upon the Denver test) and if the response was positive they were excluded from the study and referred for further investigation. Additional cases were identified from an ongoing “cohort of children”. These children were identified as potentially having CZS from those born to a cohort of pregnant women who presented with a rash (a common symptom of ZIKV infection), and from outpatient clinics of children with CZS (mostly from Oswaldo Cruz Hospital). Suspect cases were examined by a pool of specialists to confirm CZS. Data was collected between May and September, 2017.

In Rio de Janeiro, the source of the cases and controls was the Vertical Exposure to Zika Virus and Its Consequences for Child Neurodevelopment: Cohort Study in Fiocruz/IFF. Cases were children born to mothers known to be ZIKV positive, who: 1) had microcephaly, or 2) had serious developmental delay during follow-up (i.e. had Bailey score <70 between 6 and 36 months) and had other indications of CZS (e.g. eye, ear and other central nervous system abnormalities, as confirmed clinical tests and assessment by two clinicians). A second group of cases without CZS but affected by ZIKV were included in Rio de Janeiro; these cases were children who were born to ZIKV RT-PCR-positive mothers, and had mild to moderate developmental delay, as indicated by a composite Bayley score of 70–84. Control subjects were born to mothers without a history of symptoms and without developmental delay, as shown by: 1) a composite Bayley Score ≥85 conducted between 6 and 36 months following the recommended guidelines and/or 2) assessment by two paediatricians based on the child’s medical records ^28^. Data collection was undertaken between May 2017 and January, 2018.

The primary caregiver, almost always the mother, was interviewed using a structured questionnaire. In Recife, the caregivers were interviewed in their homes, at the Primary Health Centre or occasionally, in their workplace. In Rio de Janeiro the interview was undertaken in person at attendance at IFF. In Rio de Janeiro, the team comprised of a nurse, a psychologist, a social worker, and a field assistant, and they received one day training. In Recife, the interviewers had a health-related degree and previous experience in questionnaire application. They underwent 40 hours of training. The discrepancy in training time between the two sites was due to the different levels of experience of the interviewers used.

The questionnaire included items to measure social and economic impacts ( Supplementary File 2, Rio de Janeiro questionnaire). Social impact was assessed with respect to: depression, anxiety and stress, social support, family quality of life.

- The Depression, Anxiety, and Stress Scale (DASS-21) was used to assess the psychological distress among participants by investigating the symptoms of depression, anxiety, and stress ^29^. It is a 21-item questionnaire with a four-point (0–3) answer scale. The questions ask about the extent participants had experienced certain symptoms over the previous week. These items are also arranged into subscales; depression, anxiety and stress; seven items for each subscale. DASS is a reliable tool to assess psychological distress, which has been adapted and validated for Brazilian Portuguese ^30^.
- The Medical Outcomes Study Social Support Scale (MOS-SSS) is a 19-item questionnaire with each item scored on a Likert scale of 1 to 5. The scale includes five scales covering different aspects of social support (affection, positive social interaction, emotional, informational, and material). It has been validated for Brazilian Portuguese ^31^.
- Peds-QL family impact module was designed to measure the impact of pediatric chronic health conditions on parents and the family ^32^. The PedsQL™ Family Impact Module includes 36-items that encompass 6 scales measuring parent self-reported functioning: 1) Physical Functioning (6 items), 2) Emotional Functioning (5 items), 3) Social Functioning (4 items), 4) Cognitive Functioning (5 items), 5) Communication (3 items), 6) Worry (5 items), and 2 scales measuring parent- reported family functioning; 7) Daily Activities (3 items) and 8) Family Relationships (5 items). A 5-point response scale is utilized (0 = never a problem; 4 = always a problem). It was validated for Brazilian Portuguese ^33^. This question set was only asked of parents of cases.

Data was also collected on: the parents’ socio-demographic characteristics (e.g. education, asset ownership, household characteristics, income), receipt of benefits, healthcare resource consumption by child, lifecourse parameters (e.g. study, work, relationship with partner) and access and use of contraception in the postpartum period.

Two types of data were collected to be able to estimate the economic costs of CZS from the health system perspective (i.e. direct medical costs) and at the household level, which includes direct costs (e.g. travel), indirect costs (e.g. lost income because of lost work) and coping costs (e.g. interest paid on borrowed money).

In Rio de Janeiro healthcare utilization was recorded prospectively for each child in the cohort. Information included visits, hospitalization and tests for each child. For Recife, information on healthcare utilization was obtained through the questionnaire. These data were used together with the National Unified Health System (Sistema Único de Saúde [SUS]) table of procedures and their costs, which is a standardized national reference cost table based on reimbursement values by specific health service activities. Cost analysis did not include the costs of private health care.

Household-level costs were collected through the questionnaire. The initial questionnaire was designed using in-depth interviews with a sample of 4–6 women with babies who have CZS to understand their use of resources (health and social care). A questionnaire was then constructed which was administered once to all case and control caregivers in order to estimate the additional cost incurred for a child affected by CZS over a 12 month period. The questionnaire collected information on the health care and social services use, the direct medical costs (e.g. out-of-pocket expenditures), direct non-medical costs (e.g. transportation) as well as indirect costs (e.g. foregone productive work) and coping costs (e.g. selling of assets and borrowing money to deal with catastrophic expenditures).

We used the validated Brazilian Portuguese versions of the DASS 21, MOS-SSS, and PEDSQL ^30, 31, 33^. The remaining questions were written in Portuguese, and mainly taken from previously-used questionnaires ^27^. In Recife, four quantitative interviewers were included and three were included in Rio de Janeiro.

### Data entry, cleaning and storage

The database for the quantitative data from two sites was designed by the head of Informatica of Aggeu Magalhães Institute/Fiocruz, Pernambuco. The data was entered in Excel for import into the database. The software that stores the database is Microsoft SQL Server R8 and the system that reads the database was developed by the GeneXus version 10 Ev3 tool, generating the 'aspx' code for the .NET environment. Data were cleaned and merged across the two sites (Recife/Rio). Qualitative data were saved as word documents, and digital recordings. Quantitative and qualitative data were stored within the server of Aggeu Magalhães Institute/Fiocruz, Pernambuco in Brazil. All stored data were anonymized and password protected.

### Qualitative data analysis

All transcripts were transcribed by experienced transcribers and transcripts were checked by the members of the research team. Transcripts were then anonymized with codes only known to members of the research team in Rio de Janeiro and Recife. Transcripts were then shared within the entire research group. All health worker interviews and interviews with mothers were translated into English by a social scientist fluent in both English and Portuguese.

The entire qualitative research team met together in July 2017 and devised an analysis plan. The thematic analysis was undertaken independently by researchers from at least two research sites, and then were shared for feedback, modification and addition. A second workshop was then held in February 2018 to reflect on analysis and identify key themes and results to be included in papers. Feedback of results to those involved in a range dissemination events will be used as validation.

### Quantitative data analysis

New variables were recreated from the standardized questionnaires.

- DASS-21: Sub-scales were calculated for Depression, Anxiety, and Stress Scale with each subclass’s score equal to the sum of seven corresponding questions. The sum scores were multiplied by 2 in order to match the original scale score in DASS-42 so that each subscale score ranges from 0 to 42 ^29^. Categories were created for:
  ○ Depression (normal: <9; mild: 10–13, moderate: 14–20, severe: 21–27, extremely severe: >28).
  ○ Anxiety (normal: <7; mild: 8–9, moderate: 10–14, severe: 15–19, extremely severe: >20).
  ○ Stress (normal: <14; mild: 15–18, moderate: 19–25, severe: 26–33, extremely severe: >=34).
- MOS-SSS: An overall social support index was calculated, ranging from 0 to 100, with higher score indicating better availability of social support. The survey consists of one overall social support index, and four separate social support functional subscales measuring: 1) emotional/informational social support, 2) tangible social support, 3) affectionate social support, and 4) positive social interaction. MOS SSS scores were calculated as a continuous variable and analyzed on a transformed percentage scale (potential range of 0–100%) with higher scores indicative of greater support.
- PEDSQL family impact module: Items were reverse-scored and linearly transformed to a 0–100 scale (0=100, 1=75, 2=50, 3=25, 4=0), so that higher scores indicate better functioning (less negative impact) ^32^. The PedsQL Family Impact Module Total Scale Score is the sum of all 36 items divided by the number of items answered. Scale Scores were computed as the sum of the items divided by the number of items answered (to account for missing data).

Multivariable logistic regression analyses were undertaken using Stata (version 15) to compare the odds of social outcomes (i.e. maternal depression, anxiety and stress, social support, PEDSQL) comparing mothers of children with CZS to those of unaffected babies, adjusted for age, SES variables and region (Rio/Recife). Within the dataset of subjects from Rio de Janeiro, it was also possible to compare outcomes in relation to severity of developmental difficulties (assessed through the Bailey score). We stratified analyses by region (Rio/Recife) and tested for the presence of effect modification in order to assess whether there were differences in associations of these outcomes with CZS between the different settings.

### Economic data analyses and economic burden model

The economic analysis adopted the health system (SUS) and societal perspective. The health system perspective included an estimation of direct medical costs. The societal perspective included health system costs and the costs incurred by the household. As recommended in the literature, (see Drummond *et al*. 2015) when taking the societal perspective, we will provide one analysis including direct and indirect costs and one based only on the direct costs ^34^. The economic analyses calculated the incremental costs of CZS to the household, health provider and society. A subsequent burden model estimated the total economic burden of CZS in Brazil from a household, health provider and societal perspective. The different analysis sources and components for each perspective are illustrated in Table 1.

**Table 1.** Sources of data for economic analyses, from different perspectives.

Perspective	Cost component included	Data sources
Health system perspective	Direct cost of health care to the SUS. Includes costs of visits, hospitalization and tests and other items such as wheelchair or orthosis/prothesis if required.	• Database of prospective resource use of the participants combined with the SUS table of procedures and their costs • Quantitative questionnaire data
Household perspective	• Direct medical costs • Direct non-medical costs (e.g. travel) • Indirect costs (e.g. lost productivity) • Coping costs (e.g. selling assets)	• Quantitative questionnaire data
Societal perspective	Includes both, the costs to the household and the health system as above. In addition it also includes the cost of social services.	All as described above and source for cost of social services is the questionnaire

Health system cost analysis: The costs to the health system were calculated using an ingredients approach, where the number of resources (visits, days hospitalized, tests) were multiplied by the costs on the SUS table of procedures and their costs adjusted by a factor of 3.5, as suggested in the literature ^35, 36^. The data was stored in Microsoft Excel® and analyzed using R software, version 3.3.3 Costs were calculated according to the age of the child on March 31 ^st^ 2018.

Household cost analysis: The survey data were analyzed using R software, version 3.3.3 and STATA v15. We calculated the average and total additional costs of having a child with CZS at the level of the household for the 12 months prior to the questionnaire.

Societal cost analysis: The societal costs were calculated over a 12 month period as the sum of the total costs for the household, health system and social services for a baby with CZS. The additional costs of social services were estimated by analyzing the questionnaire data.

Economic modelling methods was used to estimate the community level impact of CZS on the health system, social provision and household, for the two regions. To do this, we combined our costs estimates with epidemiological data (e.g. estimates of infection prevalence, number of cases of CZS by severity) as reported by the wider Zika research community, to estimate total burden. We will present outcomes according to the health system, household and societal perspective as explained above. Older children with CZS were not available due to the recent nature of the epidemic, and so assumptions were made about the additional health and social care costs incurred by children with CZS as they become older based upon the literature for children with cerebral palsy, a similar condition. These estimates will be improved with longer term follow up and cost calculation.

We constructed an analytical model which reflects the distribution of severity of CZS cases and their associated costs, populated by projections of the total number of CZS cases. We used probabilistic sensitivity analysis (with Monte Carlo sampling), by using credible ranges and appropriate distributions for our parameters. The time horizon used for the model was 10 years, given the uncertainties in long-term costs and outcomes for these children. Outcomes will therefore be presented together with 95% confidence intervals based on percentiles. Costs were calculated in the local currency (Real) and then converted into US Dollars ($).

All analyses were additionally stratified by location (Rio/Recife) to ascertain whether there were regional differences in economic impacts.

### Data dissemination and resource sharing

Owing to the small number of children with CZS, making data potentially identifying, and the sensitive nature of the subjects discussed in the interviews, data associated with this study will not be made freely available. However, we are committed to collaborating with other researchers in the analysis of our data. Applications for access to the raw data for this study should be made by contacting Professor Hannah Kuper ( hannah.kuper@lshtm.ac.uk), Dr Tereza Maciel Lyra ( terezalyra@cpqam.fiocruz.br) or Dr Maria Elisabeth Lopez Moreria ( bebeth@iff.fiocruz.br) and outlining the purpose of the proposed analyses and the variables requested. These applications will be reviewed by the three researchers, and if accepted, the requested variables will be shared. We plan to disseminate study outcomes through the publication of open-access peer-reviewed articles.

### Ethical considerations

The ZIKV epidemic reheated discussions about abortion in Brazil ^15^. The country’s legislation strictly forbids abortion (except for cases of rape, when necessary to save a woman’s life, and anencephalic babies), and women and health care workers can be prosecuted and imprisoned for up to 3 years if they undergo or perform an abortion. Sensitive topics such as illegal abortion and other practices that go against Brazilian law and legislation were likely to arise during interviews. All data gathered remained anonymous and researchers ensured that all measures were taken so that none of the information can be tracked back to participants, protecting women and health care workers who might be involved in such practices. The data is stored on server of Aggeu Magalhães Institute/Fiocruz, Pernambuco in Brazil under password protection. Only the research team, or selected collaborators (see above for data sharing) have access to the data. Furthermore, this team includes several doctors who have the right to confidentiality in conducting interviews in Brazil (TML, TVBdA, MELM, SV) and are able to respond to external inquiry about the abortion data archive. We were also aware that we were working with women and families who may be going through extremely difficult times. Researchers who interacted with women received sensitivity training and had details of appropriate services where they could refer interviewees as needed (e.g. mental health services).

Ethical approval for the full study was received from LSHTM and the Fiocruz ethics committee (CAAE 60682516.2.1001.5269). The case-control study in Recife was approved by the Research Ethics Committees of the Pan American Health Organization (PAHO-2015-12-0075) and Fiocruz Pernambuco (CAAE: 51849215.9.0000.5190) and the Cohort study in Rio de Janeiro was approved by the IFF Ethics Committee (CAAE 52675616.0.0000.5192). The “Cohort of children” in Recife was approved by the ethics Committee of the Oswaldo Cruz University Hospital, University of Pernambuco (CAAE: 52803316.8.0000.5192). All interviewees provided written informed consent for the quantitative data collection, as did health professionals in the qualitative component. The remaining women and men interviewed qualitatively gave verbal recorded consent after an information sheet was read. The researcher signed to verify that this had been done. These precautions were taken in case information was revealed about abortion or other behaviors considered illegal in Brazil.

## Discussion and conclusions

This impact study is the first of its kind exploring the social and economic impact of ZIKV in depth. Results from the study can be used to plan services needed for mothers and other caregivers of children with CZS, and to inform future responses to epidemics.

The focus of the study was on ZIKV infection in pregnancy and CZS. The impact of ZIKV in terms of other conditions, such as Guillain-Barré syndrome, was therefore not included. The ZIKV epidemic had mostly declined by the time that the study was initiated, and so the immediate impacts were not investigated. For instance, we were not able to assess the immediate impact of the epidemic on reproductive decision making or concerns among pregnant women.

There were differences in how the data was collected in the two settings, for logistical reasons. In Recife, the interviews were conducted in the household while in Rio de Janeiro, the quantitative and many of the qualitative interviews were undertaken in the hospital or other clinical setting. This difference may influence the type and quality of information that they interviewees convey, although we tried to mitigate this issue by careful training of the interviewers, and ensuring that interviews happened in a private location where the interviews could not be overheard. Furthermore, in Recife the cases and controls were matched on health region and so would be from relatively similar socio-economic areas, which did not occur in Rio de Janeiro. We will reflect on the potential impacts of these methodological differences when discussing the results of the findings in the individual results papers.

## Data availability

No data are associated with this article.

See section “Data dissemination and resource sharing” for the data-sharing policy for the results of this study.

## Notes

^1^We had originally planned to distinguish the experience of pregnant women who tested ZIKV positive from those who tested negative. However, ZIKV infection was rare by the time we had started data collection, and reliable tests for ZIKV lacking. Furthermore, when tests were available women were not informed of their ZIKV status and there was a concern that an interviewer would inadvertently reveal the status to the pregnant woman, causing distress. We therefore did not differentiate between pregnant women by ZIKV infection status.

## Author information

Other than the three joint-first authors, all authors contributed equally and are listed in alphabetical order.

## Supplementary material

**Supplementary File 1. Interview guides for the in-depth qualitative interviews, English version.**

**Supplementary File 2. Quantitative questionnaire of the social and economic impact of Congenital Zika Syndrome, English version.**
