# Supplementary material for: Social and economic impacts of congenital Zika syndrome in Brazil: Study protocol and rationale for a mixed-methods study
Source: Wellcome Open Res. 2019 Sep 11;3:127. Originally published 2018 Oct 1. [Version 2] doi: 10.12688/wellcomeopenres.14838.2 (PMC6807146; doi:10.12688/wellcomeopenres.14838.2)
Supplement: Supplementary file 1 [file wellcomeopenres-3-16851-s0000.tgz › 4496eaf8-75eb-4387-adbc-2c1a60a59a1e_Supplementary_File_1.docx]

Appendix: Interview guides

**INTERVIEW GUIDE: MOTHERS/FATHERS AND OTHER CAREGIVERS OF BABIES WITH ZIKA**

1. Can you share the story of your/your partner’s pregnancy, from the moment you learned about the pregnancy up to the microcephaly/CZS diagnosis?
2. When you learned about the pregnancy (yours or your partner’s), what did you feel? (explore if this was a planned, wanted pregnancy)
3. Did you/your partner have pre-natal care during the pregnancy? If yes, in which health care services? How were the appointments? (explore partners/father participation)
4. Were you worried about Zika during your/your partners’ pregnancy? Why? (explore self-care strategies, care for the environment)
5. Who communicated the diagnosis of microcephaly/CZS to you? How did you feel when you received the news?
6. How did your family/partner react to the diagnosis?
7. During your pregnancy, what information about Zika did you received/looked for? (explore different sources of information). Do you think the information was sufficient/trustworthy?
8. What do you think about abortion in cases of pregnant women who had Zika? And abortion in general?
9. Could you share your child’s care routine? How do you feel about this routine? Who else is involved in this? How do they participate?
10. Could you share the support available to you (family, professionals, neighbourhs, financial support, emotional support) and that you can count on in your current situation with your child?
11. If you have other children, what are the differences in raising them and your child affected by CZS?
12. How is the relationship between siblings/other children and (name of child affected by CZS)?
13. Has your routine changed? In what ways? (explore work, routine, leisure, costs, access to government benefits).
14. What are your unmet needs?
15. Are you part of any WhatsApp group of mothers associations?
16. Would you like to complement/add anything to this interview?

**INTERVIEW GUIDE: PREGNANT WOMEN**

1. Could you share your personal history – work, study, family configuration and what are your plans for the future.
2. When you learned about the pregnancy what did you feel? What did the child’s father feel? (explore if this was a planned, wanted pregnancy)
3. How is it going, the pre-natal care during the pregnancy? Which health care services are you going to? How were the appointments with health care professionals? (explore approaches offered such as pregnant support groups, home visits by community health care workers, use of public and private services)
4. Is the father of the baby going/participating in the appointments? (explore forms of male involvement in pre-natal care and health care services support to his participation)
5. Did the Zika epidemic affected/influenced your decision to get pregnant?
6. What do you know about Zika and CZS? Do you remember the first time you heard about Zika? (explore sources and information channels)
7. Did you receive information during pre-natal care? What do you think about this information? (explore if she sees information received as sufficient or trustworthy)
8. Do you take, or have taken, measures to prevent Zika? (explore sexual transmission and self-care strategies)
9. What do you think about abortion? What about abortion in case of Zika infection? (sensitive question / be mindful of what is the best moment to ask)
10. Do you know anyone who had Zika? (explore other pregnant and children with CZS)
11. Would you like to complement/add anything to this interview?

**INTERVIEW GUIDE: WOMEN IN REPRODUCTIVE AGE**

1. Could you share your personal history – work, study, family configuration and what are your plans for the future.

2.Do you remember the first time you learned about the Zika virus?

3. Have you ever had Zika? Do you know people who had Zika? Were any of them pregnant? Do you know how the pregnancy went, the delivery and the health of both women and child?

3. After the Zika epidemic, do you identify any change in your community/neighbourhood/Family? In case this is **AFIRMATIVE**, what do you think has changed? (explore strategies of self-care, care for the environment, use of pesticide/repellent, etc)

4. Do you believe there is something that can be done to prevent Zika? (Explore what you can do and how people from your community/neighbourhood/Family can contribute).

5. Do you think you have enough information to protect yourself? Do you trust the information you have? How did you get in tough/searched this information?

6. Have you ever heard of sexual transmission of Zika?

7. Do you know women, men and/or couples who delayed pregnancy due to Zika?

8.Would you avoid pregnancy due to Zika? What is the role of men in this case? (explore contraceptive strategies)

9. What is the role of men in the case of a woman diagnosed with Zika during pregnancy?

10. How do you think you would feel if you got pregnant and had Zika?

11. What do you think about abortion? What about abortion in case of Zika infection?

12. Do you know anyone who has consider abortion in consequence of fearing Zika?

13. Do you know or have you heard about any family that had a baby with microcephaly? In case the answer is affirmative, ask: what are the challenges you heard that they face?

14. How can the public health care system help families who have had babies with microcephaly?

15. Are you aware of any help/support available to women infected by Zika?

16. Are you aware of any help/support available to families with babies affected by Zika?

17. Would you like to complement/add anything to this interview?

**INTERVIEW GUIDE: MEN IN REPRODUCTIVE AGE**

1. Could you share your personal history – work, study, family configuration and what are your plans for the future.

2.Do you remember the first time you learned about the Zika virus?

3. Have you ever had Zika? Do you know people who had Zika? Were any of them pregnant? Do you know how the pregnancy went, the delivery and the health of both women and child?

4. After the Zika epidemic, do you identify any change in your community/neighbourhood/Family? In case this is **AFIRMATIVE**, what do you think has changed?

5. Do you believe there is something that can be done to prevent Zika? Explore what you can do and how people from your community/neighbourhood/Family can contribute.

6. Do you think you have enough information to protect yourself? Do you trust the information you have? How did you get in tough/searched this information?

7. Have you ever heard of sexual transmission of Zika?

8. Do you know women, men and/or couples who delayed pregnancy due to Zika?

9.What do you think about avoiding pregnancy due to Zika? What is the role of men in this case?

10. What is the role of men in the case of a woman diagnosed with Zika during pregnancy?

11. What do you think about abortion? What about abortion in case of Zika infection?

12. Do you know anyone who has consider abortion in consequence of fearing Zika?

13. Do you know or have you heard about any family that had a baby with microcephaly? In case the answer is affirmative, ask: what are the challenges you heard that they face?

14. How can the public health care system help families who have had babies with microcephaly?

15. Are you aware of any help/support available to women infected by Zika?

16. Are you aware of any help/support available to families with babies affected by Zika?

17. Would you like to complement/add anything to this interview?

**INTERVIEW GUIDE: HEALTH CARE PROFESSIONALS**

1. What do you presently work with? Could you describe your work activities?
2. Do you remember when you first got information about Zika? (Explore timeline; how did the information reach you? By which means?)

1. Are you in direct contact with families and/or babies affected by CZS?? If **YES**, could you qualify the type of contact? (if in the health care services...)
2. Do you feel Zika has affected your work? In what ways?
3. What is your opinion about the present state of scientific knowledge in regards to Zika, comparing to the first time you learned about the vírus? (Esplore the interviewees knowledge about the subject, how is this knowledge being built, if he/she participated in any training, and how was the experience. Explore too if they were aware and instructed about sexual transmission).

1. What are your thoughts about the health services currently available to children affected by Zika, their mothers and families affected by Zika? (depending on answer, explore further the specificities of each group)
2. In regards to women who want to get pregnant, do you know how sexual and reproductive health services have been working to prevent Zika? (explore their opinions about what services should exist)
3. What do you think about services available for pregnant women (their partners and families), whether they are worried or not about Zika?
4. Still on sexual and reproductive health, what do you think about the right to abortion? (explore, afterwards, abortion in case of Zika)
5. How do you see that Zika affected:
6. Families and children?
7. Women who got pregnant at the time of sanitary emergency?
8. Women who are pregnant now? And their families (explore partners, grandparents, etc.)?
9. Women who wish to get pregnant?
10. What were the lessons brought by the Zika epidemic?
