# Supplementary material for: Social and economic impacts of congenital Zika syndrome in Brazil: Study protocol and rationale for a mixed-methods study
Source: Wellcome Open Res. 2019 Sep 11;3:127. Originally published 2018 Oct 1. [Version 2] doi: 10.12688/wellcomeopenres.14838.2 (PMC6807146; doi:10.12688/wellcomeopenres.14838.2)
Supplement: Supplementary file 2 [file wellcomeopenres-3-16851-s0001.tgz › ae1d3cca-d4f4-40b3-8c28-218348ece1f5_Supplementary_File_2.doc]

SOCIAL AND ECONOMIC IMPACTS OF CONGENITAL ZIKA SYNDROME IN BRAZIL

**SECTION 1: Identification**

1. Interviewer (ID): .......................................................................................................................... |____|____|

2. Date of interview (*ex.: 17/08/2016*): ___/ ___/ ____

3. Place of interview: 1. IFF	2. Home

3. Other health unit (please specify): ......................................................... |__| __|

4. Was the consent form signed? 1. Yes	2. No

5. Name of interviewee :

6. Name of child:

Number in cohort |____|____| Child’s medical record |______________________________|

7. What is your kinship in regards to <name>?

1. Mother ( *biological or adoptive)*	2. Father	3. Grandmother	4. Other, please specify:..............................................................

*<If not the biological/adoptive mother nor father nor legal guardian, THE INTERVIEW WILL NOT BE CONDUCTED>.*

8. Who is the main caregiver of <name>?

1. Mother	2. Father	3. Grandmother	4. Other, please specify:.........................................................................................

9. Residence address of interviewee (register *address/municipality/estate*):

10. Phone numbers *(register more than one, specifying who it belongs to):*

11. Are you presently married or living with a partner? < *the question is in regards of relationship status and not necessarily civil status >*

1. Married

2. Live together

3. has a partner but not living together

4. Not married nor living with partner presently

5. Other (Please specify): .................................................................................

6. Did not want to answer

12. You / the mother of <name> tested for ZIKA during pregnancy?

1. Yes	2. No	3. Does not know

13. *If Yes*, ask: what was the result?

1. Positive	2. Negative	3. Does not know	4. Not applicable (not tested/does not know)

**SECTION 2: Child’s information**

14. Sex: 1. Male	2. Female	3. Intersex

15. Date of birth (*day/month/year*): ____/ ___/ ____

16. Place of residence (*address/municipality/state*):

17. Tick if child belongs to group G1 or G2.

*If YES*, ask: When did you find out about <name>’s condition? (month/year)	___/ ____

If No, go to Q19

18a. Where was the final diagnosis of <name>’s condition made? <*place of diagnostic closure*>:

1. Family Health Unit

2. Basic Health Unit (Posto de Saúde)

3. Hospital, which ?: _______________________________________________________ |__|__|__|

4. IFF

5. Rede SARAH

6. Others, please specify: _____________________________________________________| __|__|__|

18b. FOR LATER CLASSIFICATION OF TYPE OF PROVIDER (POST INTERVIEW):

1. Public health unit (including hospitals)

2. Private health unit contracted by SUS

3. Non-profit health unit contracted by SUS

4. Private Hospital/ Clinic or Private Practice.

**SECTION 3: Socio-economic data**

19. Number of people with which <name> lives with (*DOES NOT include child).*

| No | Kinship to child | Age (years complete) | *Add and register number of residents* | | |
| --- | --- | --- | --- | --- | --- |
| 1 |  |  | \|___\|___\| |  | |
| 2 |  |  |  | |  |
| 3 |  |  |  | | |
| 4 |  |  |  | | |
| 5 |  |  |  | | |
| 6 |  |  |  | | |
| 7 |  |  |  | | |
| 8 |  |  |  | | |
| 9 |  |  |  | | |
| 10 |  |  |  | | |

20. What was the highest education degree achieved?

*Note: when interviewee is not the mother, ask: what is the highest education degree achieved by the mother of <name>?*

*Register number of years in formal education* (mother)|___|___|

| **PREVIOUS NOMENCLATURE** | | | **PRESENT NOMENCLATURE** | | | |
| --- | --- | --- | --- | --- | --- | --- |
| **Educational level** | **Interviewee** | **Head of household** | **Educational level** | **Interviewee** | **Head of household** | |
| 1. Illiterate or Incomplete primary education |  |  | 1. Illiterate / incomplete basic education |  | |  |
| 2. Primary/secondary complete - Junior High incomplete |  |  | 2. Fundamental I complete / Fundamental II incomplete |  | |  |
| 3. Junior high complete/High school incomplete |  |  | 3. Fundamental complete/Medium incomplete |  | |  |
| 4. High school complete/University degree incomplete |  |  | 4.Medium complete/Superior  incomplete |  | |  |
| 5. University degree incomplete |  |  | 5. Superior complete |  | |  |

21. In the house you live, how many of items listed below are there?

|  | **Quantity** |
| --- | --- |
| Television | [0] [1] [2] [3] [4 or +] |
| Radio (including any radio integrated to other devices) | [0] [1] [2] [3] [4 or +] |
| Bathrooms/toilets | [0] [1] [2] [3] [4 or +] |
| Car | [0] [1] [2] [3] [4 or +] |
| Washing machine (excluding ‘tanquinho’) | [0] [1] [2] [3] [4 or +] |
| Video/DVD any device that reads DVD, not including any car devices. | [0] [1] [2] [3] [4 or +] |
| Refrigerator | [0] [1] [2] [3] [4 or +] |
| Freezer (separate or as part of fridge) | [0] [1] [2] [3] [4 or +] |
| Computer/laptops  <do not consider tablet/smartphone/palm> | [0] [1] [2] [3] [4 or +] |
| Dish washer | [0] [1] [2] [3] [4 or +] |
| Microwave | [0] [1] [2] [3] [4 or +] |
| Motorcycle | [0] [1] [2] [3] [4 or +] |

| Clothes dryer | [0] [1] [2] [3] [4 or +] |
| --- | --- |
| Are there any domestic workers in the house? How many? | [0] [1] [2] [3] [4 or +] |

22. Where does the water in the house come from?

1. General distribution services

2. Well or spring

3. Other, which? _________________________________________________________ |__|__|

23. Consider the street you live on. You would say it is :

1. Asphalt/Paved	2. Earth/Gravel

24. Have you changed addresses after <name>’s diagnosis?

1. Yes	2. No (go to *Q 28)*

*If YES*, ask:

25. Has your monthly living expense changed? 1. Yes	2. No

26. *If YES* , ask: How much? Before: R$________	After: R$ ___________

27. Is the reason of this change of address listed below? *<accept one or more answers>*

1. (	) To be closer to health services, reducing commuting time.
2. (	) To be closer to health services, reducing expenses with commuting and food.
3. (	) To be closer to family and friends .
4. (	) Other. Please specify : ______________________________________________________ |__|__|

28. Since the diagnosis of <name> were there changes/ adaptations in your house? Consider all period after diagnosis.

1. Yes	2.No (go to *Q 30)*

29. If YES, how much did you spend with these adaptations since last year?

1. < R$ 100,00/month
2. R$ 100,00 – R$ 300,00/ month
3. R$ 300,00 – R$ 600,00/ month
4. R$ 500,00 – R$ 1000,00/ month
5. R$ 1000,00 – R$ 2000,00/ month
6. >R$ 2000,00/ month

30. During the 12 months before your child’s birth, did you work? Consider as work any activity that generated income or other compensation, even if you did it from your house.

1. Yes	2. No (go to Q 32)	2. Never worked (go to Q 32)

31. How much was your monthly income before <name>’s birth? Consider income any salary, unconditional cash tranfer (bolsa familia), internship, pension, benefit or other source. <*Individual income*>

R$	___________________

Or

1. < 1 monthly minimum wage
2. 1 monthly minimum wage
3. 1 a 2 monthly minimum wage
4. 3 a 5 monthly minimum wage
5. > 5 monthly minimum wage

6. Was not working/ never worked (non applicable)

32. Presently, you are: <read the alternatives>

1. Working? What is your occupation? ____________________________________________ |__|__|

2. Unemployed ?

3. Retired?

4. Receiving benefits? (specify): __________________________________________|__|

5. Pensioner?

6. Other (specify):_______________________________________________|__|__|

33. How much is your present monthly income? Consider what you earned in the last month, including salary, unconditional cash tranfer (bolsa familia), internship, pension, benefit or other source.

READ AND MARK TO BE ADDED AFTER END OF INTERVIEW, MARK NO WHEN INEXISTENT:

a. Salary (includes job and internship): .........................R$ ________________ (	) No
b. Pension: .................................................................... R$ ________________ (	) No
c. Benefits: .................................................................... R$ ________________ (	) No
d. Transport vouchers: ...................................................R$ _______________ (	) No
e. Bolsa família: ..............................................................R$ _______________ (	) No
f. Retirement: ................................................................R$ _______________ (	) No
g. Rents: ........................................................................ R$ _______________ (	) No
h. Other sources of income (specify): ........................... R$ _______________ (	) No

TOTAL: ......................................................................... R$ ____________

Or

1. ( ) Less than 1 monthly minimum wage
2. (	) 1 monthly minimum wage
3. (	) 1 a 2 monthly minimum wage
4. (	) 3 a 5 monthly minimum wage
5. (	) > 5 monthly minimum wage

34. When you take <name> to the health unit, do you miss a day at work?

1. Yes	2. No (go to Q 37*).*

35. *If YES*, ask: is there a loss of money because you missed work? ( *Consider here only loss related to day of work. E.g. a cleaner who could not go to work*).

1. Yes	2. No

36. How much do you lose per day, on average?

R$____________________	2. Did not know

37. What was the family income before the birth of <name>? <*Sum of all family members*>.

R$_____________________

Or

1. ( ) Less than 1 monthly minimum wage
2. (	) 1 monthly minimum wage
3. (	) 1 a 2 monthly minimum wage
4. (	) 3 a 5 monthly minimum wage
5. (	) > 5 monthly minimum wage

38. What was your family income in the last month?

R$_________

Ou

1. ( ) Less than 1 monthly minimum wage
2. (	) 1 monthly minimum wage
3. (	) 1 a 2 monthly minimum wage
4. (	) 3 a 5 monthly minimum wage
5. (	) > 5 monthly minimum wage

39. What other changes did the birth of <name> caused in your life?

<*Read slowly the alternatives bellow>*

1. Had to stop working
2. Changed jobs
3. Decreased hours worked
4. Had to stop studying
5. Had to psotpone study plans/ going back to study
6. Has more dificulty caring for other children in the
7. Has more difficulty carrying on domestic tasks
8. Think about not having other children anymore
9. There was no change.
10. Other: .......................................................................
11. Did not want to answer

40. When you take <name> to the health unit, do you pay someone else to stay in your house, either to care for other children or to execute other house tasks in your absence?

1. Yes	2. No (go to *Section 4*).

41. *If YES*, ask: How much do you pay, on average, per day?

R$__________________	2. Did not know/ could not inform

**SECTION 4: Consultations at IFF and in other health units**

42. In which of the health services <name> is treated?

List the services on table bellow:

| Name of service | Classification type of provider (*) |
| --- | --- |
| 1. IFF |  |
| 2. |  |
| 3. |  |
| 4. |  |
| 5. |  |
| 6. |  |

(*) FOR POSTERIOR CLASSIFICATION OF TYPE OF PROVIDER (POST INTERVIEWE):

1. Public health unit (including hospitals)

2. Private health unit contracted by SUS

3. Non-profithealth unit contracted by SUS

4. Private hospital / Clinic or private practice.

43. How do you go to the health units most of the time? *If necessary, mark more than one option and indicate the amount of each transport.* Consider commuting from home to the health unit and back.

|  | **Health unit**  **1:____________** | | **Health unit**  **2:_______________** | | **Health unit**  **3:______________** | | **Health unit**  **4:______________** | | |
| --- | --- | --- | --- | --- | --- | --- | --- | --- | --- |
| Which transport(s) do you use?  Insert the quantity next to each transport for the return trip | 1. By foot | ( )going  ( ) return | 1. By foot | ( )going  ( ) return | 1. By foot | ( )going  ( ) return | 1. By foot | ( )going  ( ) return | |
|  | 2. Train | ( )going  ( ) return | 2. Train | ( )going  ( ) return | 2. Train | ( )going  ( ) return | 2. Train | ( )going  ( ) return | |
|  | 3. Taxi/  Mototáxi | ( )going  ( ) return | 3.Táxi/  Mototáxi | ( )going  ( ) return | 3.Táxi/  Mototáxi | ( )going  ( ) return | 3.Táxi/  Mototáxi | ( )going  ( ) return | |
|  | 4. Private car | ( )going  ( ) return | 4. Private car | ( )going  ( ) return | 4. Private car | ( )going  ( ) return | 4. Private car | ( )going  ( ) return | |
|  | 5. IFF car | ( )going  ( ) return | 5. Carro do IFF | ( )going  ( ) return | 5. Carro do IFF | ( )going  ( ) return | 5. Carro do IFF | ( )going  ( ) return | |
|  | 6. Boat | ( )going  ( ) return | 6. Boat | ( )going  ( ) return | 6. Boat | ( )going  ( ) return | 6. Boat | ( )going  ( ) return | |
|  | 7. Bus | ( )going  ( ) return | 7. Bus | ( )going  ( ) return | 7. Bus | ( )going  ( ) return | 7. Bus | ( )going  ( ) return | |
|  | 8. Metro | ( )going  ( ) return | 8. Metrô | ( )going  ( ) return | 8. Metrô | ( )going  ( ) return | 8. Metrô | ( )going  ( ) return | |
|  | 9. Ride | ( )going  ( ) return | 9. Ride | ( )going  ( ) return | 9. Ride | ( )going  ( ) return | 9. Ride | ( )going  ( ) return | |
|  | 10.Van/  Kombi (except ones offered by city hall or health secretariat) | ( )going  ( ) return | 10.Van/  Kombi (except ones offered by city hall or health secretariat) | ( )going  ( ) return | 10.Van/  Kombi (except ones offered by city hall or health secretariat) | ( )going  ( ) return | 10.Van/  Kombi (except ones offered by city hall or health secretariat) | ( )going  ( ) return | |
|  | 11. Out-of-home treatment | ( )going  ( ) return | 11. Out-of-home treatment | ( )going  ( ) return | 11. Out-of-home treatment | ( )going  ( ) return | 11. Out-of-home treatment | ( )going  ( ) return | |
|  | 12.Other. Which? ________ | ( )going  ( ) return | 12.Other. Which? _________ | ( )going  ( ) return | 12.Other. Which? ________ | ( )going  ( ) return | 12.Other. Which? | ( )going  ( ) return |  |

44. Indicate below how much you spend, commuting to the health unit (s), the time spent commuting (Consider total commuting time from home to the health unit and back. Do not include the time you spend in the health unit. Note that extra time spent, such as taking other children to school, going to the grocery store, etc. should not enter this calculation. Add total commuting time, for example: 1 hour of one way and 2 hours of return, total of 3 hours, pertinent only to the time spent COMMUTING). Indicate also the time you remain in each health unit, quantity of trips in a month and any additional expenses.

|  | **Health Unit 1:_____________** | **Health Unit 2:_______________** | **Health Unit 3:______________** | **Health Unit 4:_____________** |
| --- | --- | --- | --- | --- |
| How much a return ticket cost? | R$ _______ going  R$ _______ return | R$ ________ going  R$ _______ return | R$ _______ going  R$ _______ return | R$ _______ going  R$ _______ return |
| How long the commute takes? | going : _________  return : _______ | going : _________  return : _______ | going : _________  return : _______ | going : _________  return : _______ |
| How many times you have to visit health units? |  |  |  |  |
| How long you stay at the health units? |  |  |  |  |
| Food | R$ _______ | R$ _______ | R$ _______ | R$ _______ |
| Gas | R$ _______ | R$ _______ | R$ _______ | R$ _______ |
| Parking | R$ _______ | R$ _______ | R$ _______ | R$ _______ |
| Other. Specify: |  |  |  |  |

45. In case you responded TFD, how do you commute to have the TFD (Tratamento Fora do Domicílio – out-of-home treatment)? Pertinent to COMMUTING only.

| 1. By foot | 7. Bus |
| --- | --- |
| 2. Train | 8. Tube |
| 3. Táxi /Mototáxi | 9. Car pool |
| 4. Private car | 10. Van (except van of health unit or governmetn) |
| 5. Boat |  |
| 6. Other, which? __________________________________________\|___\| | |

46. Besides you, who else takes <name> to the appointments? *If necessary, mark more than one option.*

1. No one (***go to Q 51)***

2. Father	3. Mother	4. Grandparent	5. Other, who? _____________________________|___|

47. *If YES, ask:* the person that goes the most with you to <name>’s appointments, work?

1. Yes	2. No

48. *If YES, ask:* does this person lose a day of work to go to the appointments with you?

1. Yes	2. No

49. *If YES, ask :* does this person lose money because he/she didn’t go to work? (*Consider only loss per day not worked, e.g a cleaner who misses payment of the day).*

1. Yes	2. No

50. *If YES, ask :* How much money does he/she loses per day on average? R$ _______________

51. In regards to **<name>’s appointments**, how frequently does he/she goes to each specialist? *<name>’s consultations only.*

| **Professional** | **Health Unit 1:____________** | | **Health Unit 2:_____________** | | **Health Unit 3:_____________** | | **Health Unit 4:______________** | |
| --- | --- | --- | --- | --- | --- | --- | --- | --- |
| **Frequency**  **(W – Weekly/M- Monthly)** | **W** | **M** | **W** | **M** | **W** | **M** | **W** | **M** |
| Cardiologist |  |  |  |  |  |  |  |  |
| Dentist |  |  |  |  |  |  |  |  |
| Speech Therapist |  |  |  |  |  |  |  |  |
| Physiotherapist |  |  |  |  |  |  |  |  |
| Geneticist |  |  |  |  |  |  |  |  |
| Infectologist |  |  |  |  |  |  |  |  |
| Nephrologist |  |  |  |  |  |  |  |  |
| Neurologist |  |  |  |  |  |  |  |  |
| Nutricionist |  |  |  |  |  |  |  |  |
| Ophtalmologist |  |  |  |  |  |  |  |  |
| Orthopedist |  |  |  |  |  |  |  |  |
| Otorhinolaryngology |  |  |  |  |  |  |  |  |
| Paediatrician |  |  |  |  |  |  |  |  |
| Psychologist / Psychiatrist |  |  |  |  |  |  |  |  |
| Ocupational therapist |  |  |  |  |  |  |  |  |
| Urologist |  |  |  |  |  |  |  |  |
| Other: |  |  |  |  |  |  |  |  |
| Other: |  |  |  |  |  |  |  |  |
| Other: |  |  |  |  |  |  |  |  |

**SECTION 5: Events in the last 12 months related to hospitalizations**

52. Has <name> been hospitalized anytime after his birth?

1. Yes	2.No (go to section ***6*).**

53. *If YES, ASK:* How many times? |___|___|

99. Not applicable (in case of no hospitalizations)

54. *If YES, ask:* for how long was <name> hospitalized for in the last time? |___|___| days

55. During last hospitalization, did someone from your family, friend or anyone else needed to stay in your house to help with domestic activities?

1. Yes	2. No (***go to question 57*).**

56. *If YES, ask:* Did you have to pay this person to stay at your house?

1. Yes, how much? R$ _______________________

2. No

57. Has someone else stayed with <name> during hospitalization? *If YES, who?*

*Mark more than one option if necessary.*

1. No one (***go to question 61*).**

2. Father	3.Mother	4. Grandparent	5. Other, who? ____________________ |__|__|

58. *If YES, ask:* this person who also stayed with <name> during hospitalization, works?

1. Yes	2. No (***go to section 6***).

59. *If YES, ask:* Does he/she missed a day of work to stay with <name> in the hospital?

1. Yes	2. No

60. *If YES, ask:* How much this person lost, on average, per day?

R$____________________________	2. Did not know

61. During <name>’s last hospitalization, what was the average expenditure?

Write bellow the expenses related to last hospitalization:

| **Type of expense** | **Total spent on each hospitalization (R$)** |
| --- | --- |
| Parking |  |
| Transport |  |
| Gas |  |
| Printing |  |
| Food |  |
| Hospital fees |  |
| Diagnostic exams fees |  |
| Other: |  |

**SECTION 6: Questions related to treatment expenses**

62. Did yuou need to borrow money at any point due to <name>’s condition?

1. Yes	2. No (***go to question 66*).**

63. *If yes*, how much?: R$_________________

64. Do you pay/have paid interest due to this loan?

1. Yes	2. No

65. *If yes*, how much?: R$_________________

66. Does <name> have individual private health insurance?

1. Yes	2. No (***go to question 68*)**

67. *If yes*: How much do you pay for his/her individual private health care insurance? <*Atentiono: do not consider any value payed torwards other family member* >.

1. < R$ 500,00	2. R$ 500,00 - RR$ 1000,00	3. > R$ 1000,00

68. Do you pay someone on a regular basis to help with house chores, care for other children, or other daily tasks?

1. Yes	2. No

*If yes*, how much per month?: R$_________________

69. After <nome>’s birth, what other extra expenses did you started to have due to his condition? ? *(Specify frequency – monthy (M), weekly (W), only once (O))*

Fill table bellow: <*Do not include expenses already included previously>.*

| ITEM | R$ | Frequency |
| --- | --- | --- |
| Medication and vitamins |  |  |
| Exams |  |  |
| Food |  |  |
| Spectacles |  |  |
| Hearing device |  |  |
| Other: |  |  |

| **Module 2 - Reproductive health and contraception** | |
| --- | --- |
| 70. Name of interviewee: | |
| 71. When you found out you were pregnant, you: *(mark only one)*  1. were glad  2. accepted pregnancy  3. considered abortion  4. Was confused, did not know what to do.  5. Other. Which? ___________________________ \|__\| | |
| 72. In regards to your partner, how much has your relationship changed since <name>’s birth: (mark only one)  1. Nothing changed 2. Separated you  3. United you 4. He was not my partner  5. Other. Which?__________________________ \|__\|__\|  6. Does not know/Does not remember  7. Did not want to answer | |
| 73. After <name>’s birth, did you receive any information regarding family planning / how to avoid or delay pregnancy?  1. Yes 2. No (go to Q 76) 3. Does not remeber | |
| 74. Where did you get the information from?  1. In the maternity where <name> was born  2. At a PSF Unit or home via community health care agent  3. Other (specify): .................................................................. \|__\|___\| | |
| 75. Who gave you the information?   1. Physician 2. Nurse 3. Midwife | 4. Social worker 5. Someone else. Who?_____________ \|__\| 6. Does not know 7. Not applicable |
| 76. Were you prescribed contraception?  1. Yes 2. No (*go to* Q 79) | |
| 77. Were you asked about your preference for contraceptive method?   1. Yes 2. No 8. Not applicable | |
| 78. Were you informed about where to get contraception?   1. Yes 2. No 3. Not applicable | |
| 79. Do you use presently any contraceptive method to avoid pregnancy?  1. Yes 2. No  Explore: what about your partner?..........................................................................  *If YES*, ask: Which? .......................................................................................... | |

In addition, standardised scales:

- MOS Social Support Survey (19 item questionnaire)
- Depression Anxiety and Stress Scale 21 (DASS21 – 21 item questionnaire)
- Pediatric Quality of Life (PEDSQL) – Family Impact Module (20 item questionnaire)
