# Supplementary material for: Social and economic impacts of congenital Zika syndrome in Brazil: Study protocol and rationale for a mixed-methods study
Source: Wellcome Open Res. 2019 Sep 11;3:127. Originally published 2018 Oct 1. [Version 2] doi: 10.12688/wellcomeopenres.14838.2 (PMC6807146; doi:10.12688/wellcomeopenres.14838.2)
Supplement: Peer review history

# Peer review: Reviewer response for version 2

**DOI:** 10.21956/wellcomeopenres.16851.r36441

**Published:** 2019-10-22

**Authors:** Anne C. Wheeler

**Affiliations:** 1 RTI International, Research Triangle Park, NC, USA

Thank you for your thorough response to my concerns. Although I still maintain that a protocol paper written in the past tense is confusing, I feel all of my other concerns have been addressed and this is a minor issue that if the other reviewers and editor feels is not a problem I am ok on that point. I believe this is ready for indexing and will make an important contribution to the field.

I confirm that I have read this submission and believe that I have an appropriate level of expertise to confirm that it is of an acceptable scientific standard.

# Peer review: Reviewer response for version 1

**DOI:** 10.21956/wellcomeopenres.16167.r35634

**Published:** 2019-06-26

**Authors:** Anne C. Wheeler

**Affiliations:** 1 RTI International, Research Triangle Park, NC, USA

This paper describes a rationale and protocol for assessing the social and economic impacts of congenital Zika syndrome in Brazil. The goals are important and the methods seem sound. I was confused that the entire paper is written in the past tense given this is describing a protocol without any results. It might be better to write in the future tense if describing only the protocol. Alternatively, describe the protocol within a results-driven set of papers.

 More information about how the different sets of information from the two sites will be synergy for the analysis is needed.  

 More information about the Denver test is warranted and how these results will be compared with the Bayley results from the other site

 It is unclear how the state/government economic information was collected.

I confirm that I have read this submission and believe that I have an appropriate level of expertise to confirm that it is of an acceptable scientific standard, however I have significant reservations, as outlined above.

# Response

**Published:** 2019-08-14

**Authors:** Hannah Kuper

**Affiliations:** Clinical Research Unit, Department of Infectious and Tropical Disease, London School of Hygiene and Tropical Medicine, UK

Response to Dr Wheeler

 Thank you for the detailed and thoughtful comments on the manuscript, which we have attempted to address, as follows:

 1.     Changing tense of paper: We would prefer to retain the past tense, as we specified that the data collection has already been completed and so believed that it was confusing to then write in the present tense. We would prefer to retain a separate protocol paper, as otherwise it would not be possible to present all the methods for the entire study together (as results paper will be quantitative, qualitative or economic) and to present the level of detail desired due to the word restrictions on journal submissions.

 2.     More information has been provided on how data was combined between the two sites, and how data analyses were stratified by region and included tests for effect modification to ascertain whether there were different patterns in the two sites.

 3.     3-4 age appropriate questions, selected from The Denver test, were used to screen potential control subjects in Recife to ascertain whether they appeared to have developmental delays which could be indicative of Congenital Zika Syndrome. The full Denver test was not undertaken. These results were not collected on the other subjects (cases Recife, cases and controls Rio). The Bayley result was only collected within Rio. We therefore do not have the possibility of comparing the Denver Test and the Bayley results. This process is now clarified in the text.

 4.     For the economic analyses, we included costs from the health system, household and societal perspective as specified in Table 1. State/government economic information was not collected.

 Best wishes,

 Hannah and colleagues

# Peer review: Reviewer response for version 1

**DOI:** 10.21956/wellcomeopenres.16167.r34013

**Published:** 2018-11-07

**Authors:** Debora Diniz

**Affiliations:** 1 Law School, University of Brasília, Brasília, Brazil

The study is original and fills a gap in the literature about the legacy of Zika affected countries, such as Brazil. The methodology is complex and well designed, combining mixed methods of social sciences, such as qualitative interviews and survey. My general analysis is very supportive of the study and my comments are more related to inspire the authors during the analysis process than any specific criticism to the design. I thank the authors for the opportunity of reading the piece in advance.

 1. There are two settings in the study, a potential source which is not fully explained how they will inspire the analysis. I have two concerns related to the settings: a) the study mentions "contrast" between them - I wonder how do the authors plan to contrast the settings and based on which categories? b) there was an important difference in how the data was collected in setting 1 to the setting 2- the first one the interviews were made at women's houses; the second one the interviews were made at the hospital. There are a lot of evidences showing how the space alters the quality of the evidence. Also how to compare/contrast the two settings considering the space changing? Besides that, there is also sufficient evidence showing that women feel themselves constrained of talking with researchers at hospital settings. How to deal with that? I wonder if we do not have two different studies using the same protocol, but they cannot be compared at the end. How to answer that point?

 2. There is a description about the team who performed the interviews. But there is no clarification about the gender and nationality - were they women and Brazilians? Locals or from different regions? Again - talking about women's issues, like reproduction, care or family, is mostly a woman to woman conversation and it matters a lot the cultural identification of the researcher. There is no clarification about that in the study.

 3. Page 3 states that the major focus of the response was vaccines, diagnosis, etc. I'm not sure about that, if you consider the local/regional response. The major response was to eliminate the vector. I'd suggest the authors to clarify the context of the statement, as it matters to understand participant's narratives to the political response.

 4. The focus of the study is the concept of family. Rarely, the authors mention "women" or the "caregiver". This is my main concern about the study: what is the sociological and political concept of family for a study like this? Who is the family? A lot of studies about Zika and about child's care in Brazil show that it's a woman's role and responsibility. To talk about family has two consequences: a) the most important one is to put woman in the shadow about the legacy of Zika and her role on caring; b) the second one is to ignore the composition of welfare state in Brazil. Despite of the fact that there has been huge changes in the government and how social policies have been protecting these women, the focus has to be more on the weak social state than on the family's role to guarantee child's needs and protection. Expressions like "families directly affected" does not sound much related to Brazilian culture/reality - "women directly affected in their care roles/as bread winners" would be more appropriate. I do not know much about what is the concept of the family and the family composition of the participants by the paper.

 4. There are moments in the study that the authors use "microcephaly" as the same as CZS. I would suggest to padronize. 

 5. There is a reference to a study (note 25) estimating the medical costs of a Zika affected individual during her lifetime. I'm not sure about the reliability of citing a study like that in which medical treatment is the central variable in a social science study. To calculate costs like that we have to include: a) woman with no paid work; b) changes in the welfare state and the reforms on cash benefit transfers or other social benefits; c) educational inclusion or not. My point is that if the authors want to make an economic impact of Zika it has to be more comprehensive than that.

 6. How the economic data was collected? 

 7. Why did the authors mention "contrast" between the settings? How to make any comparison if there was a change in the protocol interviewing women at the hospital and women at home? And how different were the two setting to describe it as "contrast"?

 8. How to justify the difference of time used for training between the two groups? Why did it happen?

 9. Finally I really appreciated the careful measures taken to protect women's identity and confidentiality related to abortion. Abortion in Brazil is against the law and there is to confidentiality protection to researchers. I have some questions to improve the women's protection: a) Were the records destroyed? b) Who have now access to the women's records? c) Is there a journalist in the team? If yes, she/he should be the  the gate keeper of the raw data or the one to respond to any external inquiry about the abortion data archive. Journalists are the only one with right to confidentiality in conducting interviews in Brazil; d)  Where is the storage of the material? If the researchers need more understanding about the ethical and legal questions about this issue, I'm more than happy to collaborate.

I confirm that I have read this submission and believe that I have an appropriate level of expertise to confirm that it is of an acceptable scientific standard.

# Response

**Published:** 2019-08-14

**Authors:** Hannah Kuper

**Affiliations:** Clinical Research Unit, Department of Infectious and Tropical Disease, London School of Hygiene and Tropical Medicine, UK

Dear Dr Diniz,

 Thank you for the detailed and thoughtful comments on the manuscript, which we have attempted to address, as follows:

 1.     We have now clarified in the methods that in order to contrast the two regions, the quantitative analyses were stratified by region, with formal testing for the presence of effect modification. Data collection methods differed between the two sites, as you correctly noted, which is now clarified as a limitation in the discussion with consideration for how this issue was mitigated as far as possible and dealt with in the analysis and reporting of the results papers.

 2.     We have clarified in the text the interviewers were all female Brazilian social scientists from the local region.

 3.     We have emphasised that the major local/regional response was the elimination of the vaccine, while national/international efforts also focussed on vaccines, diagnosis, etc.

 4.     We have revised the text throughout to place greater emphasis on the social and economic impacts on women/mothers, and have largely replaced “family” with “household”.

 We have now used the term CZS throughout, unless microcephaly was expressly meant, we tried to clarify in the text how these conditions were different.

 5.     We agree that a focus on medical costs alone is insufficient. Therefore, in our data collection we have included costs of direct costs (e.g. travel or medical costs), indirect costs (e.g. lost productivity) and coping costs (e.g. need to borrow money and pay interest), as now clarified in Table 1 and in the description of quantitative data collected. The economic modelling will use a time horizon of 10 years, since there are great uncertainties in the long-term costs and outcomes for these children (e.g. potential impact of educational exclusion or welfare changes).

 6.     As described in point 5, we have attempted to provide more information on how economic data was collected.

 7.     As clarified in point 1, we have tried to explain how we “contrast” the two settings and take account for the differences in context of interviews in interpreting the findings.

 8.     The time of training of interviewers differed between the sites because of differences in the experience of those recruited.

 9.     We have included additional information on how we attempted to preserve the anonymity and protect women who may have made statements about abortion during the interview, including through anonymization and safe storage of data, restrictions on data sharing, and the inclusion of doctors in the team who can be gate-keepers for the data. We would, of course, value your expertise in navigating these difficult issues.

 Best wishes

 Hannah and colleagues
